# Temperature dependent structure and dynamics in smectite interlayers: ^23^Na MAS NMR spectroscopy of Na-hectorite†

**DOI:** 10.1039/c9ra01056d

**Published:** 2019-04-25

**Authors:** Raju Nanda, Geoffrey M. Bowers, Narasimhan Loganathan, Sarah D. Burton, R. James Kirkpatrick

**Affiliations:** Department of Chemistry, Michigan State University East Lansing MI 48824 USA; Department of Chemistry, Bar-Ilan University Ramat Gan Israel 52900 nanda.raju85@gmail.com nanda.raju@biu.ac.il; Department of Chemistry and Biochemistry, St. Mary's College of Maryland St. Mary's City MD 20686 USA; William R. Wiley Environmental and Molecular Sciences Laboratory, Pacific Northwest National Laboratory Richland WA 99352 USA; Department of Earth and Environmental Sciences, Michigan State University East Lansing MI 48824 USA

## Abstract

^23^Na MAS NMR spectroscopy of the smectite mineral hectorite acquired at temperatures from −120 °C to 40 °C in combination with the results from computational molecular dynamics (MD) simulations show the presence of complex dynamical processes in the interlayer galleries that depend significantly on their hydration state. The results indicate that site exchange occurs within individual interlayers that contain coexisting 1 and 2 water layer hydrates in different places. We suggest that the observed dynamical averaging may be due to motion of water volumes comparable to the dripplons recently proposed to occur in hydrated graphene interlayers (Yoshida *et al. Nat. Commun.*, 2018, **9**, 1496). Such motion would cause rippling of the T-O-T structure of the clay layers at frequencies greater than ∼25 kHz. For samples exposed to 0% relative humidity (R.H.), the ^23^Na spectra show the presence of two Na^+^ sites (probably 6 and 9 coordinated by basal oxygen atoms) that do not undergo dynamical averaging at any temperature from −120 °C to 40 °C. For samples exposed to R.H.s from 29% to 100% the spectra show the presence of three hydrated Na^+^ sites that undergo dynamical averaging beginning at −60 °C. These sites have different numbers of H_2_O molecules coordinating the Na^+^, and diffusion calculations indicate that they probably occur within the same individual interlayer. The average hydration state of Na^+^ increases with increasing R.H. and water content of the clay.

## Abstract 2

^23^Na MAS NMR spectroscopy of the smectite mineral hectorite acquired at temperature from −120 °C to 40 °C shows the presence of complex dynamical processes in the interlayer galleries that depend significantly on their hydration state.

## Graphical abstract

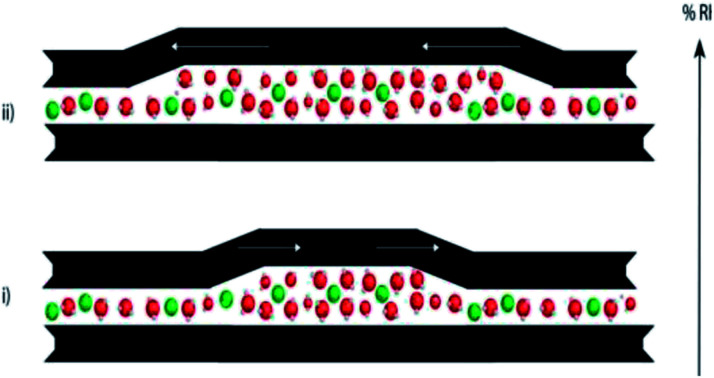

## Introduction

The interactions among ions, water, and a substrate are fundamental processes in fields as diverse as biology, medicine, chemistry, materials science, geology, and space science. In many of these fields, and especially materials science and the geosciences, the substrate for the interactions is often a solid oxide or hydroxide material which can have wide ranging structural and chemical properties. Thus, there has been substantial effort devoted to understanding the structural, dynamical, and energetic aspects of the interaction of ions and water with geochemically important minerals^1–28^ and materials of technological importance.^29–32^ The temperature and pressure dependence of these interactions is complex and has been a long standing topic of discussion.^33–38^ The smectite group of clay minerals has been of particular interest in geoscience, chemistry and materials science, because of their abundance and ability to intercalate a wide variety of organic and inorganic cations, along with water and other fluid species. Because of their remarkable swelling capacity, they have been of long-standing interest in nuclear waste management, in composites with polymers and in 2D-nanomaterials.^37,39–42^

Because the ions and molecules in the interlayer galleries of smectite clays are structurally and dynamically disordered, their molecular scale behavior is difficult to study with diffraction methods, and in recent years experimental spectroscopic methods and computational molecular modeling have been widely employed.^1,2,5,7–12,43^ This paper reports the results of an experimental, variable temperature ^23^Na nuclear magnetic resonance (NMR) and computational molecular dynamics (MD) modeling study of the structural and dynamical behavior of Na^+^ in the widely investigated smectite clay, hectorite. The ^23^Na NMR behavior of hectorite has been studied previously,^1^ but the results here provide more comprehensive understanding, because the spectra were obtained at a higher H_0_ field strength (850 MHz ^1^H frequency) and over a wider range of temperatures and water contents. The MD modeling results provide important insight into the molecular scale behavior of the interlayers and support the structural and dynamical interpretations of the NMR results. Hectorite is commonly used in NMR studies of smectites, because its low Fe content greatly reduces paramagnetic line broadening compared to other natural smectites such as montmorillonite.^44^ Hectorite containing a wide variety of exchangeable species has been studied by NMR methods,^1,8,9,22,23,44^ X-ray diffraction (XRD),^1,45–48^ thermogravimetric analysis (TGA),^1,44,46^ and quasi-elastic neutron scattering (QENS).^24–26,49–51^ In parallel, there have also been several computational molecular modeling studies of it using principally MD methods.^10,14,16,21,25,43,49,52^

The results here show complex, temperature dependent dynamical behavior that depends greatly on the water content of the sample. We interpret this complex behavior to be due to diffusional motion in individual interlayer galleries with varying local hydration states and, thus, varying interlayer thicknesses (basal spacings) along their length. Such behavior requires the tetrahedral-octahedral-tetrahedral (T-O-T) layers of the clay to be flexible and dynamic. The basal XRD reflections of smectites are commonly quite broad, which is usually interpreted to be due to coexisting interlayers with different basal spacings, rather than variable spacings within individual interlayers. However, as discussed in detail below there is growing evidence that smectite T-O-T layers are flexible and dynamic, and thus that their interlayer galleries could be comparably dynamic.^11,12,33–38^

## Materials and methods

### Materials

The hectorite sample used in this study was the standard San Bernardino hectorite (SHCa-1), which is available from the Source Clays Repository of the Clay Minerals Society. This hectorite develops its permanent structural charge from Li^+^ for Mg^2+^ substitution in the octahedral sheet of the T-O-T layers and contains only a trace amount of paramagnetic iron, making it useful for the NMR studies.^1,9,44^ The charge balancing cation in the as-received sample is predominantly Na^+^, with lesser amounts of K^+^, Mg^2+^, and Sr^2+^. It's permanent structural layer charge is −0.35 on an O_10_ basis (−0.70 on an O_20_ basis), and the structural formula of the as-received sample is (Na^+^_0.19_Mg^2+^_0.07_Sr^2+^_0.01_K^+^_0.01_)^0.36+^[(Mg^2+^_2.65_Li^+^_0.35_)^0.35−^(Si_3.99_Al_0.01_)O_10_(F_1.1_H_0.9_)].^44^ The samples used for NMR studies underwent a multistep Na-exchange process, as described in our earlier reports.^1,53^ Prior to NMR data acquisition, these Na-exchanged samples were exposed to relative humidities (R.H.s) of 0%, 29%, 43%, 70% and 100%. The 0% R.H. sample was held in a vacuum oven at 50 °C for 24 hours, and the 100% R.H. sample was held in a desiccator over deionized water for 30 hours. The 29%, 43%, and 70% R.H. samples were held in desiccators over saturated solutions of different salts^54^ for 30 hours. The extents of H_2_O adsorption were determined by weighing samples before and after exposure to the different R.H.s, and the H_2_O/Na molar ratio was calculated by assuming 0.35 Na^+^/formula unit (given above). These values are listed in Table 1.

**Table 1:** The absorbed water content and the H_2_O/Na^+^ molar ratio of Na-hectorite exposed to different R.H.s

R.H. (%)	Water content (g per f.u.)	H_2_O/Na^+^ molar ratio
0	0.0003 (0.000017)	0.40
29	0.0009 (0.000049)	1.17
43	0.0019 (0.000105)	2.50
70	0.0065 (0.000361)	8.60
100	0.0137 (0.000760)	18.10

### ^23^Na MAS NMR methods

The temperature dependent ^23^Na Bloch-decay NMR experiments were performed at a resonance frequency of 224.77 MHz under magic angle spinning (MAS) conditions at a spinning frequency of 10 kHz using an 850 MHz Agilent DDR2 solid state NMR spectrometer located at the Environmental and Molecular Science Laboratory of the Pacific Northwest National Laboratory. The spectra were collected with 500 transients using a spectral width of 100 kHz, and the spectra contain 2048 data points.

A pulse delay of 1.5 to 5 s was used depending on the temperature. The chemical shifts of all the spectra were referenced to a 1 M NaCl(aq) solution that was also used for the pulse width calibration. Power was adjusted to produce a liquid-state π pulse of 10 μs. A quantitative and central-transition-selective pulse width of 0.45 μs was used to obtain the spectra of the clay samples, since ^23^Na is a spin 3/2 quadrupolar nucleus. All spectra were processed using MestreNova software and received one zero fill. An exponential apodization of 100 Hz was used for the 40 °C, 20 °C, 0 °C, −20 °C and −40 °C spectra, and 1000 Hz was used for the −60 °C, −80 °C, −100 °C, and −120 °C samples. Samples were equilibrated for 30 min at each temperature prior to NMR data acquisition. The temperature was controlled using a standard Varian VT stack temperature controller with a liquid nitrogen cooled VT gas. Samples were packed in 4 mm, thick wall zirconia NMR rotors sealed at both ends with a rubber cap in order to prevent water loss during data acquisition. All the samples were weighed before and after the experiment to evaluate water loss during the NMR data acquisition and showed negligible water loss (±0.2%). All the spectra were analyzed using the iterative fitting program DMfit with an estimated fitting uncertainty of ±1%.^55^

### Computational methods

The simulated hectorite model has a structural formula of Na_0.4_(Mg_2.6_Li_0.4_)Si_4_O_10_ (OH)_0.94_F_1.06_, in close agreement with the composition of the natural sample.^1,18^ The F^−^ atoms are located in two different nearest neighbor environments: (i) coordinated to three Mg and (ii) coordinated to two Mg and one Li. The ratio of these two sites is 3/2, in accordance with site intensities from ^19^F NMR spectra of the natural sample.^56^ There is no tetrahedral Al^3+^ for Si^4+^ substitution in this model, since the natural sample contains a negligible fraction of such sites.

The simulation supercell contains 2 interlayer regions and consists of 32 unit cells of hectorite (4 × 4 × 2) with lateral dimensions of approximately *L*_*x*_ = 21.0 Å and *L*_*y*_ = 36 Å. The size of the model allows for a quasi-disordered distribution of the Li^+^/Mg^2+^ substitution sites following generalization of Lowenstein's rule.^57^ The Li^+^/Mg^2+^ and F^−^/OH^−^ distributions were assigned only after building the supercell to avoid the possibility of repetitive patterns in different T-O-T layers. For each simulation, all the Na^+^ ions were initially placed at the mid-plane of the interlayers. Twenty six different models were investigated with water contents ranging from 0 to 24H_2_O molecules/Na^+^ ion. The water contents used in our simulation model will permit us to probe a range of hydration states, starting with collapsed interlayers and up to 4- layer hydrates. As a result, these simulated models will be able to predict different hydration states observed experimentally at different R.H. conditions. We performed classical MD simulations in the NPT and NVT ensembles at ambient conditions (*T* = 300 K and *P* = 1 bar) using the LAMMPS simulation package.^58^ A Nose–Hoover thermostat and barostat were used to control the temperature and pressure independently in all 3 directions.^59,60^ Three-dimensional periodic boundary conditions were employed. A cutoff distance of 10 Å was applied for the short-range non-electrostatic interactions, and Ewald summation technique was used to compute long-range electrostatic interactions. A time step of 1 fs was employed in our simulations. Each system was equilibrated for 5 ns followed by a production run for another 2 ns in NPT ensemble. The MD trajectories were recorded every 10 fs during production run for the analysis of thermodynamic properties. The potential stable hydration states were determined based on the thermodynamic analysis from NPT simulations. The interlayer structural and dynamic properties were then obtained subsequently by running simulation in the NVT ensemble for these hydration states. These simulations were run for 5 ns for equilibration and an additional 2 ns for data production where the trajectories are recorded every 10 fs for structural interpretation. Our previous papers provide further details about this hectorite model and the simulation and analysis methods.^11,12,61,62^

## Results

### Experimental results

**Fig. 1:**
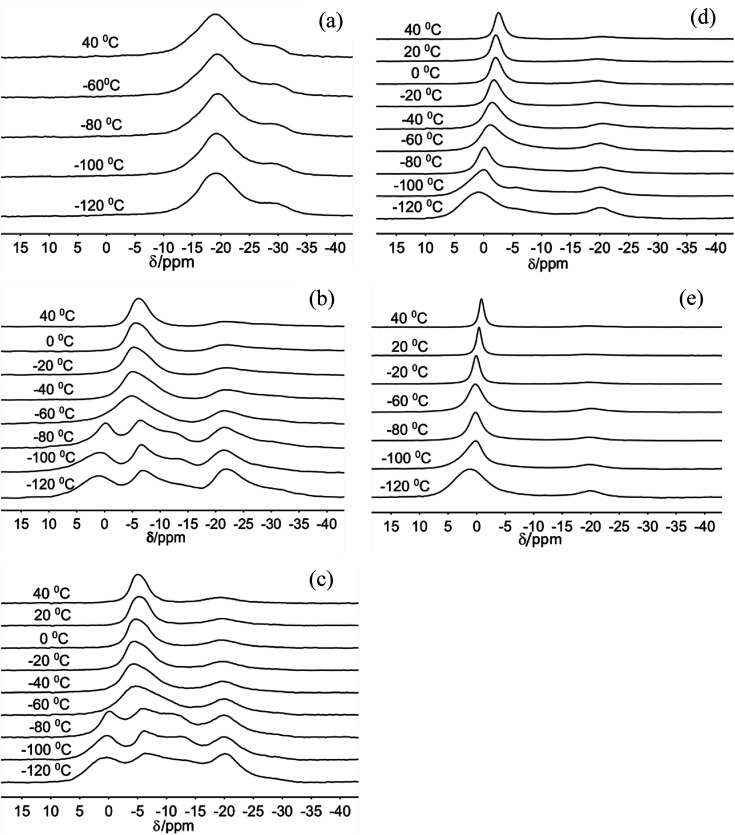
^23^Na MAS NMR spectra of Na-hectorite exposed to 0% R.H. (a), 29% R.H. (b), 43% R.H. (c), 70% R.H. (d), and 100% R.H. (e) acquired at the indicated temperatures.

**Fig. 2:**
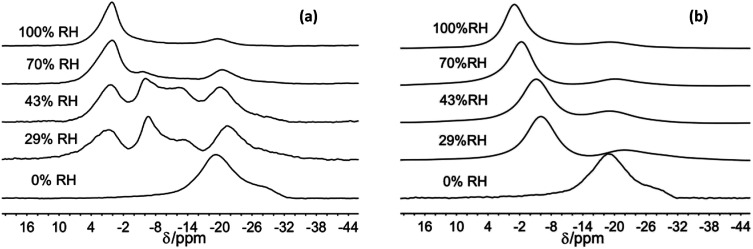
^23^Na MAS NMR spectra of Na-hectorite exposed to the indicate R.H.s at −100 °C (a), and 40 °C (b).

The ^23^Na MAS NMR spectra of Na-hectorite show great variation with R.H. and temperature (Fig. 1, 2, Table S1, and Fig. S1†). At 0% R.H. there are two resonances centered at ∼−29 ppm and ∼−19 ppm at all temperatures from −120 °C to 20 °C. The peak maxima and relative intensities of these two resonances do not vary significantly with temperature, and the relative intensity and FWHH of the resonance at ∼−29 ppm are less than those of the −19 ppm at all temperatures. The spectra of the samples exposed to 29% and 43% R.H. vary greatly with temperature. At −120 °C, −100 °C, and −80 °C they contain five resonances, which coalesce to three at higher temperatures (Fig. 1b and c). At 29% R.H. the two most shielded resonances at ∼−21 and −27 ppm resemble the ∼−19 and −29 ppm resonances observed at 0% R.H. and are observable above the baseline at all temperatures. At 43% R.H. the most shielded resonance at ∼−27 ppm is not resolvable above the baseline except at −120 °C, where it occurs at −27.4 ppm, and the resonance at ∼−19 ppm does not change significantly with temperature. For both the 29% and 43% R.H. samples at −120 °C, −100 °C, and −80 °C there are three other, more deshielded resonances with peak maxima of ∼−11 ppm, ∼−6 ppm and ∼0 to 1 ppm. For the 29% R.H. sample, from −120 °C to −80 °C these resonances become generally narrower (especially the one at ∼0 to 1 ppm), and at −60 °C they merge into a single, symmetrical resonance centered at ∼6 ppm. With increasing temperature this resonance narrows and becomes more symmetric, and its peak maximum becomes more shielded (more negative). The behavior of the 43% R.H. sample with increasing temperature is generally similar, except that from −60 °C to 0 °C fitting of the merged peak requires two resonances.

For the 70% and 100% R.H. samples at −120 °C there are three resolvable resonances centered at ∼1.2 to −2.7 ppm, ∼−6 ppm, and ∼−20 ppm. The resonance at ∼−29 ppm observed at low temperatures and lower R.H.s is not resolvable above baseline for these samples, and the position, FWHH and relative intensity of the ∼−20 ppm resonance does not change significantly with temperature. At 70% R.H. the width of the most deshielded resonance at ∼0 ppm decreases from −120 °C to −80 °C, and at −60 °C it merges with the ∼−6 ppm resonance into an asymmetrical peak. At higher temperatures, this peak becomes narrower and more symmetrical, and its peak maximum becomes more shielded (more negative). At 100% R.H. the two most deshielded resonances centered at ∼−1 and −5 ppm at −120 °C merge into a single asymmetrical resonance at −100 °C which becomes more symmetrical, narrower (except at −60 °C), and more shielded (more negative) with increasing temperature. Fig. 2 highlights the greatly different effects of varying temperature and R.H. on the ^23^Na NMR spectra. For instance, at low temperature (−100 °C) there is progressive loss of signal intensity for the ∼−6 and −12 ppm resonances with increasing R.H. (Fig. 2a), and at high temperature (40 °C) the merged peak in the ∼ −4 to 2 ppm range becomes progressively more shielded (Fig. 2b).

### Computational results

**Fig. 3:**
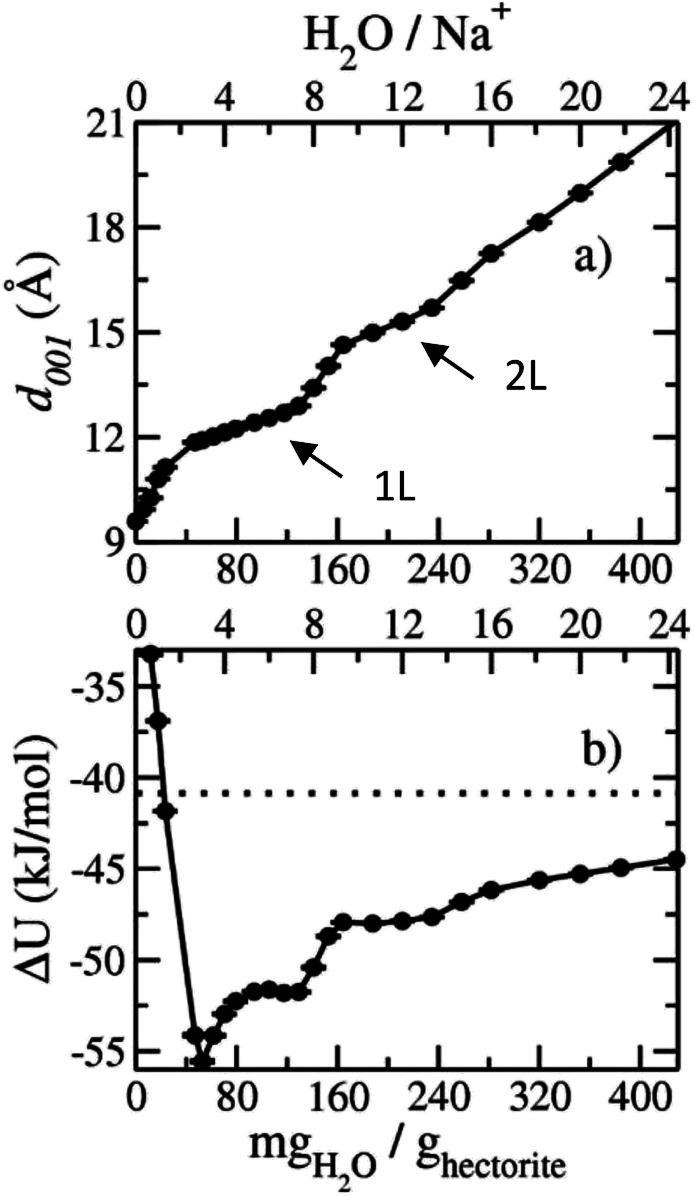
Computed swelling properties of Na-hectorite with a molar F^−^/(F^−^ + OH^−^) ratio of 0.53 as functions of interlayer H_2_O content: (a) basal spacing; (b) hydration energy. The horizontal dashed line in (b) indicates the internal energy of the bulk SPC water model used in the simulations.

The computed interlayer spacings of the model Na-hectorite increase with increasing water content and show well-defined monolayer (1L) and bilayer (2L) hydrates, along with continuous expansion at higher hydration states (Fig. 3a). The basal spacing of 9.5 Å for the collapsed (0H_2_O) model is in good agreement with the experimental value of 9.7 Å.^1^ The 1L and 2L hydrates are characterized by distinct plateau regions at basal spacings of ∼12.0 Å and 15.0 Å, respectively and is consistent with the experimental values.^1^ For both the 1L and 2L states, however, the computed basal spacings increase slightly with increasing water content. The computed basal spacings for the dry and 1L and 2L hydrates here are very close to those calculated for Na-hectorite with no F^−^ for OH^−^ substitution,^10^ suggesting that the macroscopic expansion behavior of hectorite is not greatly affected by the F^−^/OH^−^ ratio.

**Fig. 4:**
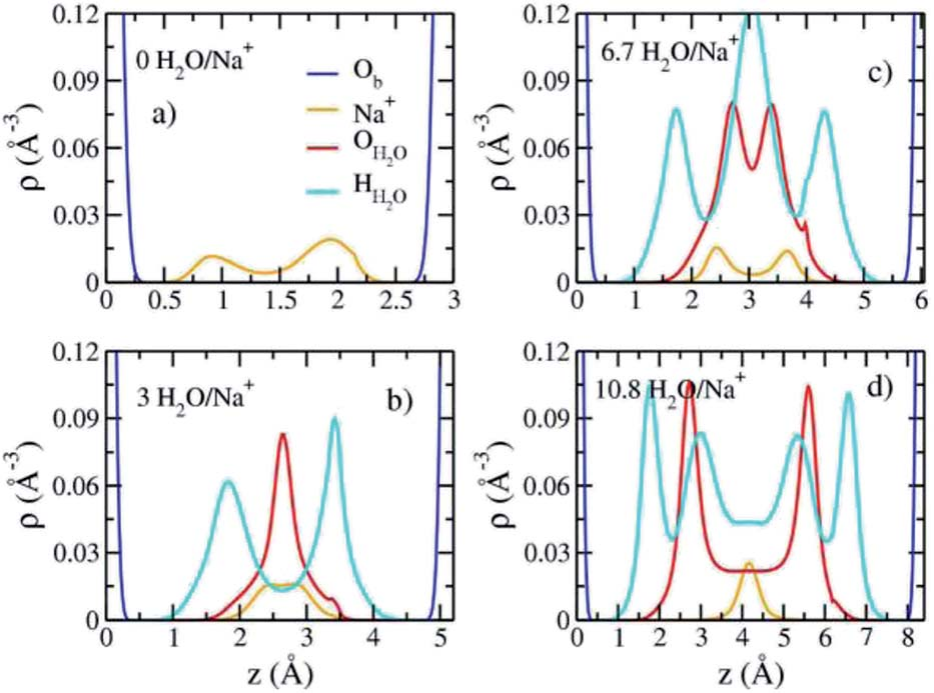
Computed atomic density profiles (ADPs) as a function of distance normal to the basal hectorite surface (*z* (Å)) for the indicated interlayer species in Na-hectorite with a molar F^−^/(F^−^ + OH^−^) ratio of 0.53 at the indicated H_2_O/Na^+^ ratios. (a) collapsed structure; (b) least hydrated monolayer structure; (c) fully hydrated monolayer structure; (d) bilayer structure.

The computed interlayer hydration energies for Na-hectorite show three minima at H_2_O/Na^+^ ratios of 3.0, 6.7 and 10.8 with values of −56, −52 and −48 kJ mol^−1^, respectively (Fig. 3b). The minima at H_2_O/Na^+^ = 3.0 and 6.7 coincide with the beginning and complete formation of a 1L hydrate structure with basal spacings that increase from 12.0 Å to 12.6 Å. The broader and shallower minimum centered at H_2_O/Na^+^ = 10.8 corresponds to the 2L hydrate with varying water contents and shows that the hydration energy does not vary much with H_2_O/Na^+^ ratio in this range as the basal spacing increases from about 14.8 Å to 15.6 Å. Although the computed energies are similar to those for Na-hectorite with no F^−^ for OH^−^ substitution,^10^ the H_2_O/Na^+^ ratios are somewhat higher here, because the smaller layer charge of our model (−0.8 |*e*| *vs.* −1.0 |*e*| in the earlier study) requires fewer Na^+^/formula unit, leaving more interlayer space for H_2_O adsorption. The computed atomic density profiles (ADPs) for the interlayer species in the simulations with H_2_O/Na^+^ = 0, 3, 6.7, and 10.8 (Fig. 4) are generally similar to the comparable profiles for Na-hectorite with no F^−^ for OH^−^ substitution,^10^ except at H_2_O/Na^+^ = 3, which has the global minimum energy (Fig. 3). The ADP of Na^+^ for this composition is characterized by a broad distribution near the center of the interlayer (Fig. 3b), indicating constant ion hopping between sites closer to one or the other basal surface. The H_2_O molecules are located in the midplane of the interlayer region and are represented by a single peak for O_H_2_O_ and 2 peaks for H_H_2_O_.

These results support the interpretation of experimental XRD results for fluorohectorite that indicate that the Na^+^ ions are located on two different positions closer to one or the other basal surface and that H_2_O molecules are at the midplane of the interlayer gallery.^46^

The absence of two well resolved ADP peaks for Na^+^ in our studies is probably due to the presence of ∼50% OH^−^ as opposed to the 100% F^−^ composition studied by Kalo *et al.*^46^ In this case, the Na^+^ is located at the center of a ditrigonal cavity of one basal surface and above a Si tetrahedron of the other (Fig. 6c). The details of the RDFs and RCNs at higher hydration states are similar to those for Na-hectorite with no F^−^ for OH^−^ substitution.^10^ This result indicates that F^−^ for OH^−^ substitution has progressively less effect on the interlayer cations with increasing distance from the basal surface.

**Fig. 5:**
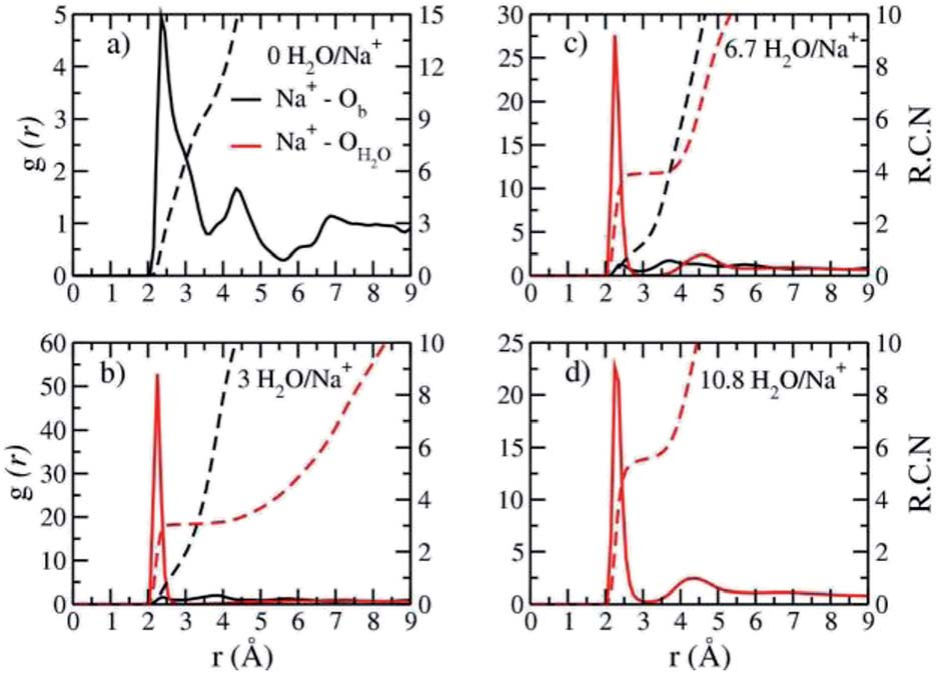
Computed radial distribution functions (RDFs – (*g*(*r*)) is defined as the probability of finding atoms O_b_, O_H_2_O_ around Na^+^ as a function of distance between them) and corresponding running coordination numbers (RCNs) of Na-hectorite with a molar F^−^/(F^−^ + OH^−^) ratio of 0.53 at the indicated H_2_O/Na^+^ ratios. (a) collapsed structure; (b) least hydrated monolayer structure; (c) fully hydrated monolayer structure; (d) bilayer structure.

**Fig. 6:**
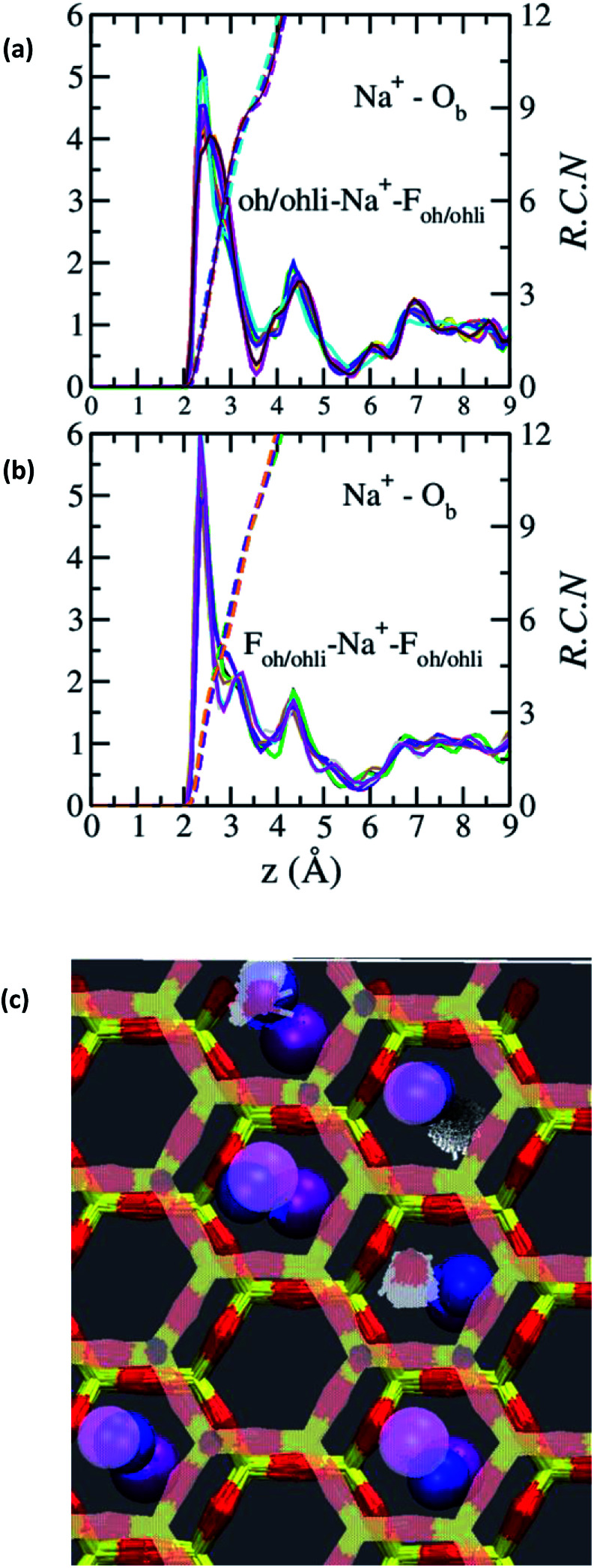
RDFs and RCNs between Na^+^ and O_b_ in the collapsed (0H_2_O) interlayer of Na-hectorite with a molar F^−^/(F^−^ + OH^−^) ratio of 0.53. (a) Na^+^ near an OH^−^ of one bounding T-O-T layer and an F^−^ of the other. (b) Na^+^ near an F^−^ on both bounding T-O-T layers. (c) Schematic representation of these two different environments with many trajectories superimposed on top of each other. Hexagonal structure = Si and O_b_ of the basal surfaces (top surface in pale colors). Blue balls = Na^+^. Purple balls = F^−^ (top surface in pale colors). Small pale pink objects = O of OH^−^, white = H of OH^−^.

The Na^+^ ADP for Na-hectorite with no F^−^ for OH^−^ substitution,^10^ shows a single, narrow peak near the center for the interlayer O_b_, that H_2_O molecules partially hydrate them, and that they also coordinate the O_b_ atoms. The computed radial distribution functions (RDFs) and associated running coordination numbers (RCNs) of Na^+^ show substantially different coordination environments at different hydration states (Fig. 5a–d). For the collapsed (0H_2_O) structure the mean nearest neighbor interatomic distance between Na^+^ and O_b_ is ∼2.4 Å with a shoulder at ∼3.0 Å (Fig. 5a). This distribution is significantly different from that for Na-hectorite with no F^−^ for OH^−^ substitution, which has a well resolved peak at 2.5 Å and does not have the shoulder at 3.0 Å.^10^ These differences are due to differences in the coordination environments of Na^+^ ions near an F^−^ on both sides of the interlayer and those near an F^−^ on one side and an OH^−^ on the other (Fig. 6a and b). There is a negligible number of Na^+^ near OH^−^ on both sides. The nearest neighbor O_b_–Na^+^ RCN for Na^+^ near two F^−^ sites is 6, 3 from each basal surface. That for Na^+^ near one F^−^ and one OH^−^ is 9, 6 from one side and 3 from the other.

## Discussion

### Structural interpretations

The two temperature-independent ^23^Na NMR resonances at ∼−29 and ∼−19 ppm for the 0% R.H. Na-hectorite sample that are also present with decreasing abundances at higher R.H.s (Fig. 1) represent rigidly held interlayer Na^+^. Since the molar H_2_O/Na^+^ ratio of the 0% R.H. sample is 0.40 (Table 1) and some of this water is probably on external particle surfaces, we interpret these resonances to be due to Na^+^ coordinated by only O_b_ atoms of the basal surfaces with little coordination by the oxygen atoms of water molecules (O_w_). Similar behavior has been observed for ^133^Cs in Cs-hectorite.^44^ The NMR chemical shifts of such nuclides as ^23^Na, ^133^Cs, ^27^Al, and ^29^Si in oxide systems are well known to become more shielded (more negative) with increasing nearest neighbor (NN) coordination number.^44^ For these two resonances, the ∼10 ppm difference in the peak maxima indicates significantly different NN coordination numbers. The MD simulations for fully dry Na-hectorite here show Na^+^ with either 6 and 9 NN O_b_. Thus, we interpret the ∼−19 ppm resonance to be due to Na^+^ in dominantly 6-fold coordination by O_b_ and ∼−29 ppm resonance to Na^+^ in dominantly 9-fold coordination by O_b_, with the specific structural environments discussed above (Fig. 6a–c). These coordinations are very different than the 9- and 12-fold coordinations proposed for Cs^+^ in Cs-hectorite^44^ due to the smaller size of Na^+^.

The presence of the ∼−19 ppm resonance at all R.H.s and temperatures shows that our Na-hectorite contains interlayers in which the Na^+^ is not significantly coordinated by H_2_O molecules under any of the experimental conditions. The spectral fitting results (Table S1†) indicate that at 29% and 43% R.H. approximately 35 to 40% of the Na^+^ is in such environments and that at 100% R.H. this amount is in the 10–16% range. The loss of the ∼−29 ppm resonance at R.H.s of 43% and greater suggests that some water is entering these interlayers and causes the Na^+^ ions to preferentially occupy 6-coordinate sites. Similarly, the decreased relative intensity of the ∼−19 ppm resonance with increasing R.H. indicates that progressively more of these interlayers become hydrated as the H_2_O activity (R.H.) increases.

The appearance of the resonances in the low temperature spectra near 0, −6 and −12 ppm at R.H.s greater than 0% clearly demonstrates that they are due to Na^+^ coordinated to at least some O_w_. The resonance at ∼0 ppm is readily assigned to Na^+^ coordinated by 6 O_w_. Na^+^ in 6-coordination in aqueous solutions occurs near 0 ppm,^54,63–65^ and NaCl in 1 M solution is the chemical shift standard for 0 ppm. This interpretation is supported by the known deshielding of Na^+^ with increasing coordination by water,^1,66^ and the observations that this resonance is the most deshielded one observed and dominates at 100% R.H. Steric considerations require that 6-fold coordination of Na^+^ by H_2_O in smectites must occur in interlayers that are at least 2L hydrates, and our MD simulations show that all the Na^+^ in 2L layers in Na-hectorite have this coordination by O_w_ (Fig. 5d). The presence of both the ∼−19 and 0 ppm resonances at R.H.s of 29% and more, thus, clearly demonstrates the coexistence of interlayer environments with greatly different hydration states with significant amounts of H_2_O present.

The presence of the resonances near −6 and −11 ppm observed at low temperatures at R.H.s of 29% and greater and their progressive decrease in relative intensity at larger R.H. demonstrates that they must be due to interlayer Na^+^ coordinated to fewer than 6 O_w_ along with some number of O_b_. Given the observation of coexistence of 0L and 2L states in our samples and the previously reported coexistence of at least 1L and 2L states in Na-hectorite at 43% R.H.^1^ at least one of these resonances must be due to Na^+^ in 1L hydration. Our MD simulations for 1L interlayers shows that the average NN coordination of Na^+^ is 3.0 O_w_ and 2.0 O_b_ at H_2_O/Na^+^ = 3.0 and 4 O_w_ and 1 O_b_ at H_2_O/Na^+^ = 6.7 and that the interlayers are disordered. Since coordination to O_w_ causes deshielding relative to coordination to O_b_ (*e.g.*, the NN coordinations of the ∼0 ppm and ∼−19 ppm resonances), we assign the ∼−11 ppm resonance to Na^+^ with 3.0 O_w_ and 2.0 O_b_ nearest neighbors and the ∼−6 ppm resonance to Na^+^ with 4 O_w_ and 1 O_b_ nearest neighbors. These assignments are in agreement with the progressive decrease in the relative intensity of the ∼−11 ppm peak compared to that of the ∼−6 ppm peak with increasing R.H. The interlayers are, of course, highly disordered, and other coordinations are also like to be present at lower abundances. Collectively then, the ^23^Na NMR spectra of our samples in combination with the MD modeling results demonstrate that in the presence of water Na-hectorite contains multiple coexisting hydration states at a given R.H. and that the average hydration of the Na^+^ ions increases with increasing R.H. The relatively small energy differences between the different hydration states (Fig. 3b) supports the argument that they can be expected to coexist, especially given the potential for different local compositions of the T-O-T layers themselves.

### Dynamical interpretations

The temperature dependence of the ^23^Na NMR spectra demonstrates that the rate of dynamical exchange of Na^+^ among the structural environments represented by the resonances centered near ∼0, ∼−6, and ∼−11 ppm increases with increasing temperature and provides some constraint on this rate. In NMR spectra, dynamical site averaging causes observable merging of peaks if the frequency of exchange is greater than approximately 0.1× the difference in peak positions in frequency units, with full merging occurring at frequencies ∼10× this difference.^44^ Dynamical effects can also cause narrowing of a single peak due to averaging of chemical shift anisotropy (CSA), second order quadrupolar broadening or static structural disorder. Both effects occur in our samples. The presence of the ∼0, −6 and −11 ppm peaks at −80 °C and below demonstrates that there is no exchange of Na^+^ between these environments at frequencies >∼0.25 kHz (0.1 × 11 ppm × 224 Hz ppm^−1^). With increasing temperature, these peaks merge into one that becomes progressively narrower with increasing temperature. In a three-site exchange model the chemical shift of the averaged peak (*δ*_avg_) is given by

1 *δ*_avg_ = *δ*_1_*I*_1_ + *δ*_2_*I*_2_ + *δ*_3_*I*_3_

where *δ*_*i*_ and *I*_*i*_ (*i* = 1, 2, 3) represent the observed chemical shifts and average fractional intensities of the respective resonances. Comparison of the observed chemical shifts of the resonance in the 0 to −6 ppm range for the 40 °C spectra with that calculated values assuming complete dynamical averaging of the three low temperature peaks ∼0, −6 and −11 ppm shows very good agreement (Table 2), clearly demonstrating that this resonance is the average of the three low temperature ones. The appearance of multiple resonances in the ^133^Cs NMR spectra of Cs-hectorite at low temperature and their peak averaging at higher temperature supports this argument.^44^ With increasing R.H. the sum of the relative intensities of the ∼0, −6 and −11 ppm peaks at low temperatures and the averaged peak at 40 °C increase and the sum of intensities of the ∼ −19 and −27 ppm decrease (Table S1†) indicating that progressively more of the interlayers are being hydrated and that all the Na^+^ in the hydrated interlayers is participating in the dynamical averaging. The progressively more deshielded (less negative) position of the dynamically averaged peak at 40 °C with increasing R.H. is due to averaging among sites with progressively more NN O_w_.

**Table 2:** Calculated average peak maxima (*δ*_avg_) assuming 3-site averaging of the three most shielded Na^+^ resonances at −120, −100, and −80 °C compared to the observed value of the dynamically average resonance at 40 °C for Na-hectorite exposed to he indicated R.H.s

R.H. (%)	*T* (°C)	*δ* ppm (fractional RI)			*δ*_avg_ (ppm)	*δ* (ppm at 40 °C)
29	−120	1.1(0.5)	−6.6(0.3)	−11.9(0.2)	−4.0	−6.4
	−100	1.1(0.6)	−6.7(0.2)	−11.7(0.2)	−3.4	
	−80	−0.1(0.6)	−6.5(0.2)	−11.4(0.2)	−4.6	
43	−120	1.0(0.4)	−6.3(0.3)	−12.0(0.3)	−5.3	−5.3
	−100	0.6(0.4)	−6.3(0.2)	−11.6(0.4)	−5.4	
	−80	−0.1(0.4)	−5.8(0.4)	−11.1(0.2)	−5.4	
	−60	−4.2(0.2)	−6.3(0.8)		−6.0	
	−40	−4.0(0.4)	−7.0(0.6)		−5.7	
	−20	−4.1(0.4)	−6.7(0.6)		−5.7	
	0	−4.4(0.4)	−6.5(0.6)		−5.7	
70	−120	1.1(0.6)	−6.2(0.4)		−1.8	−2.7
	−100	0.4(0.6)	−6.0(0.4)		−2.3	
	−80	−0.2(0.6)	−5.5(0.4)		−2.2	
	−60	−1.2(0.5)	−3.9(0.5)		−2.5	
100	−120	1.2(0.8)	−5.6(0.2)		−0.1	−0.9

It is not possible to uniquely model the frequencies of this site exchange at each temperature due to uncertainty related to potential quadrupolar and CSA effects. However, the widths of the averaged peaks at −60 °C are approximately half the full range of the chemical shifts at low temperature, indicating that the frequency of the site exchange process is of the order of 2.5 kHz at this temperature. The decreasing width of the averaged peak with increasing temperature shows that this exchange frequency progressively increases, but it is not possible to uniquely calculate the values. The decreasing width of the averaged peak at 40 °C with increasing R.H. suggests that this frequency increases with increasing water content and average hydration state of the Na^+^.

The narrowing of the individual resonances with increasing temperature in the −120 °C to −80 °C range demonstrates an increasing frequency of dynamical averaging within the NN environments of Na^+^ (*e.g.*, cage rattling) without rapid exchange among the different environments. This is clearest for the ∼0 ppm resonance for Na^+^ coordinated by 6 water molecules (Fig. 1). Such line narrowing is expected, because the activation energy for motion of the Na^+^ ions relative to their NN H_2_O molecules is expected to be much less than that for Na^+^ or H_2_O site exchange. The interplay of this nearest neighbor motion and site exchange is clearly illustrated for the ∼0 ppm peak at 70% and 100% R.H. From −120 °C to −80 °C this resonance narrows, but intensity for the less hydrated sites remains resolvable. At −60 °C, however, the resonance in this region broadens due to the exchange among these sites, and at higher temperatures it narrows again due to more rapid site exchange.

The molecular scale mechanism of the observed site exchange is likely to involve both motion of the H_2_O molecules and the Na^+^ ions. MD simulations of Na-hectorite with no F^−^ for OH^−^ substitution show that the diffusion coefficients of H_2_O molecules are somewhat larger than those of Na^+^ ions in both 1WL and 2WL interlayers, but that they are of the same order of magnitude (10^−7^ cm^2^ s^−1^ for 1L and 10^−6^ cm^2^ s^−1^ for 2L hydrates near room temperature).^10^ For these values, characteristic diffusion distances (∼[*Dt*]^1/2^) on the ms NMR time scale are of the order of 0.1 μm for 1L and 0.3 μm for 2L hydrates.^10^ These distances are much more than enough to cause local averaging within a homogeneous interlayer at room temperature. The diffusion coefficients at −60 °C, the lowest temperatures at which site exchanges are observed, are not known but are certainly much less than at room temperature. The characteristic diffusion lengths are, thus, expected to be much less than at room temperature. The lateral dimensions of the individual particles of the hectorite we use are of the order of 1 to a few μm, larger than the characteristic diffusion lengths at room temperature and certainly larger than at −60 °C. This result suggests that site exchange between separate 1L and 2L interlayers would not occur rapidly enough to cause the observed changes in the NMR spectra. We conclude, then, that the NN coordination environments of Na^+^ giving rise to the ∼0, −6 and −11 ppm resonances at low temperature all occur within individual interlayers, and thus that the hydration state at a specific position in an individual interlayers varies with time.

For the observed site averaging to occur within in individual interlayer, the basal spacing of that interlayer must dynamically expand and contract as the Na^+^ and H_2_O diffuse relative to the T-O-T layers and each other. This requires that the T-O-T layer bend and ripple with an out-of-phase component to the power spectrum. The ability of the T-O-T layers of clays and micas to bend has been reported in many experimental and computational studies. For instance, MD simulations of the smectite mineral beidellite show reversible (elastic) bending due to an external stress of about <0.7 GPa.^33^ Similar elastic behavior has been reported for muscovite mica at external stresses as high as 2 GPa.^33^ A combination of MD modeling and AFM and TEM observations for clays and mica show that this bending occurs by bond stretching and bending and causes rearrangement of the alkali metal ions on the surfaces of the layers.^38^ Large-scale MD simulations of smectite clays show dynamical undulatory behavior.^35,36^ There are also other reports showing bending of clay layers in polymer composites.^37^ Cryo-TEM images of montmorillonite in water show bent T-O-T layers and variable interlayer thickness in the osmotic swelling regime, demonstrating flexibility of the T-O-T layers under these conditions.^67^ To our knowledge there have been no large-scale computational studies of smectites with partially hydrated interlayers that might be expected to show dynamic fluctuations in local interlayer hydration state in addition to undulatory behavior. MD studies of water in graphene, however, do show dynamical rippling of the C-sheets and the coexistence of 1L and 2L water domains within individual interlayers.^68^ In these simulations, the 2L domains (called dripplons by the authors) move rapidly within the interlayer gallery during the 1 ns simulation time and cause bending of the graphene sheets. The dripplons are stable to the addition of solute and show rapid lateral motion at rates comparable to that of the diffusion of individual water molecules. Large scale MD studies of layered double hydroxides also show rippling of the metal hydroxide layers and small fluctuations of the thickness of individual interlayers.^69^ For such behavior to cause complete averaging of the ∼0, −6 and −11 ppm resonances in our ^23^Na NMR spectra, this frequency must be greater than approximately 25 kHz, the range of chemical shifts of the averaged peaks. This frequency is orders of magnitude slower than the calculated rate of motion of dripplons in graphene. MD simulations of very large smectite-water systems at a range of temperatures and experimental approaches such as fluid cell TEM^67^ will be needed to explore this issue further.

## Conclusions

Variable temperature ^23^Na NMR spectra of variably hydrated Na-hectorite in combination with computational MD modeling shows the coexistence of several local hydration states of Na^+^ in an individual sample and that the average hydration state increases with increasing R.H. NMR resonances for these different hydration states are observable at temperature of <−80 °C. At higher temperatures they merge due to dynamical site exchange, and the results show that this must occur within individual interlayers. This dynamics must result in rippling of the T-O-T layers as the interlayers expand and contract, as recently proposed for hydrated graphene.^68^ At 0% R.H. Na^+^ has NN coordinations of 6 and 9 all temperatures, and the relative intensities of these peaks decrease with increasing R.H as the interlayers become hydrated. Na^+^ ions on these sites do not participate in the dynamical averaging. At R.H.s of 29% and greater there are additional resonances that we assign to Na^+^ with nearest neighbor coordinations of 3.0 O_w_ and 2.0 O_b_, 4 O_w_ and 1 O_b_, and 6 O_w_. Na^+^ with these coordinations coexist within individual interlayers and participate in the dynamical averaging. Their average hydration state increases with increasing R.H.

## Conflicts of interest

There are no conflicts to declare.

## Supplementary Material

RA-009-C9RA01056D-s001
