# Supplementary material for: Temperature dependent structure and dynamics in smectite interlayers: 23Na MAS NMR spectroscopy of Na-hectorite
Source: RSC Adv. 2019 Apr 25;9(22):12755–65. doi: 10.1039/c9ra01056d (PMC9063695; doi:10.1039/c9ra01056d)
Supplement: RA-009-C9RA01056D-s001 [file RA-009-C9RA01056D-s001.pdf]

Electronic Supplementary Material (ESI) for RSC Advances.
This journal is © The Royal Society of Chemistry 2019

Temperature dependent structure and dynamics in smectite interlayers: ^{23}Na MAS NMR spectroscopy of Na-hectorite

Raju Nanda[1,2], Geoffrey M. Bowers[3], Narasimhan Loganathan[1], Sarah D. Burton[4], R. James Kirkpatrick[1,5]

[1]Department of Chemistry, Michigan State University, East Lansing, MI, 48824, USA

[2] Present address: Department of Chemistry, Bar-Ilan University, Ramat Gan, Israel, 52900

[3]Department of Chemistry and Biochemistry, St. Mary's College of Maryland, St. Mary's City, MD, 20686, USA

[4]William R. Wiley Environmental and Molecular Sciences Laboratory, Pacific Northwest National Laboratory, Richland, WA, 99352, USA

[5]Department of Earth and Environmental Sciences, Michigan State University, East Lansing, MI, 48824, USA

Supporting Information

Table S1. Fitted peak maxima, full-widths at half-height (FWHH), and relative peak intensities of ^{23}Na MAS NMR spectra of Na-hectorite collected at the indicated temperatures and relative humidities (R.H.s).

T **(^{0}C)**	**δ (ppm)**	**FWHH (ppm)**	**Relative Intensity/%**
	**0% R.H.**		
40	-19.1	8.7	92.8
	-28.7	5.4	7.2
-60	-19.4	7.9	86.2
	-29.0	6.6	13.8
-80	-19.5	7.8	87.7
	-29.2	6.7	12.3
-100	-19.4	7.8	86.4

	-29.3	6.9	13.6
-120	-19.3	8.2	85.8
	-29.3	6.5	14.2
	**29% R.H.**		
40	-6.4	4.0	64.6
	-21.7	4.7	8.1
	-26.8	13.5	27.4
0	-6.1	4.9	63.2
	-21.9	4.8	10.1
	-27.5	12.2	26.7
-20	-5.9	5.6	71.6
	-21.8	5.6	11.7
	-28.3	12.6	16.7
-40	-5.9	6.6	61.8
	-21.9	4.8	10.8
	-27.4	12.6	27.5
-60	-5.2	8.3	72.7
	-21.6	5.2	14.1
	-28.1	10.8	13.2
-80	-0.1	4.1	30.8
	-6.5	4.0	14.7
	-11.4	6.5	15.5
	-21.7	5.9	19.2
	-28.4	12.0	19.9
-100	1.1	7.5	38.0
	-6.7	3.5	9.5
	-11.7	7.5	16.7
	-21.6	5.7	21.6
	-28.3	9.7	14.2
-120	1.1	7.6	26.2
	-6.6	4.8	21.5
	-11.9	8.2	9.8
	-21.8	5.7	21.3
	-27.2	12.9	21.3
	**43% R.H.**		
40	-5.3	3.4	73.0
	-19.4	6.7	27.4
20	-5.5	4.0	65.8
	-19.6	6.7	34.2
0	-4.4	2.8	26.7
	-6.5	3.7	45.3
	-19.7	6.5	28.0
-20	-4.1	3.2	27.8
	-6.7	4.2	44.0
	-19.8	6.4	28.2
-40	-4.0	4.0	31.8

	-7.0	5.5	40.2
	-19.8	6.2	28.0
-60	-4.1	4.0	8.6
	-6.3	9.9	53.0
	-19.9	6.9	38.4
-80	-0.1	4.1	22.3
	-5.8	5.2	22.2
	-11.1	5.9	18.0
	-20.0	7.2	37.1
-100	0.6	5.8	25.5
	-6.3	4.1	15.0
	-11.6	6.8	22.6
	-20.4	6.7	37.0
-120	1.0	7.0	26.3
	-6.3	5.5	17.3
	-12.0	8.7	22.3
	-20.6	6.1	33.2
	-27.4	7.0	1.0
**70% R.H.**			
40	-2.7	1.9	76.2
	-21.1	6.2	23.8
20	-2.2	2.0	83.9
	-19.8	6.5	16.1
0	-2.2	2.4	79.8
	-19.4	5.5	20.2
-20	-2.0	2.9	74.2
	-19.7	6.0	25.8
-40	-1.7	3.9	74.4
	-21.1	6.2	25.6
-60	-1.2	3.8	36.2
	-3.9	9.6	35.0
	-20.0	6.0	28.9
-80	-0.2	2.7	42.5
	-5.5	11.4	26.1
	-20.0	6.4	31.4
-100	0.4	5.0	45.2
	-6.0	6.0	32.0
	-20.1	5.2	22.8
-120	1.1	6.6	50.8
	-6.2	7.5	33.9
	-20.0	5.3	15.3
**100% R.H.**			
40	-0.9	1.1	82.9
	-20.1	6.0	17.2
20	-0.4	1.1	83.6
	-19.8	6.3	16.4

-20	0.1	1.5	85.9
	-19.9	5.1	14.2
-60	0.2	3.3	83.9
	-20.3	5.1	16.2
-80	0.2	2.7	82.2
	-19.7	5.1	17.8
-100	0.4	4.0	84.1
	-19.7	5.1	15.9
-120	1.2	6.5	70.9
	-5.6	7.3	17.2
	-19.9	5.0	12.0

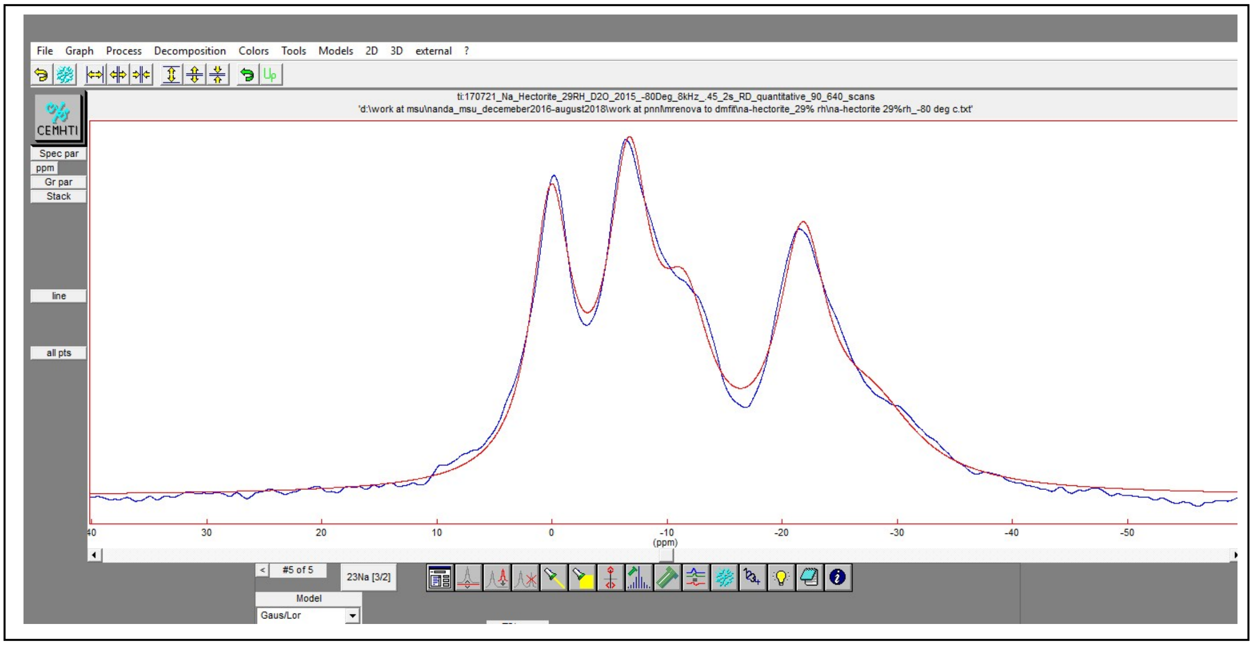

Figure S1: A representative example of the fitting of the ^{23}Na MAS NMR spectra discussed in this paper using the DMfit. This spectrum is for Na-hectorite exposed to 29% R.H. at -80^{0}C and if fit with five Lorentzian components. The blue and red lines are the experimental and fitted spectra, respectively.
